# Case Report: Clinically amyopathic dermatomyositis presenting acutely with isolated facial edema

**DOI:** 10.12688/f1000research.13604.2

**Published:** 2018-03-26

**Authors:** Efthymia Pappa, Marina Gkeka, Asimina Protogerou, Leonidas Marinos, Chariclia Loupa, Constantinos Christopoulos

**Affiliations:** 1 Department of Internal Medicine, Amalia Fleming General Hospital, Athens, 15127, Greece; 2 Department of Hematopathology, Evangelismos General Hospital, Athens, 10376, Greece

**Keywords:** dermatomyositis, amyopathic dermatomyositis, inflammatory myopathy, edema

## Abstract

A 45-year-old Asian man presented with acute-onset periorbital and facial edema associated with pyrexia. Muscle weakness was absent. Initial laboratory investigations showed an inflammatory reaction, while screening for infections was negative. Serum muscle enzyme levels were normal. He was hospitalized and treated empirically with antibiotics and corticosteroids, pending the result of facial skin and muscle biopsy. He showed a good clinical and laboratory response but an attempt to discontinue corticosteroids led to a prompt relapse of facial edema and pyrexia, associated with rising laboratory indices of inflammation. Biopsy findings were typical of dermatomyositis. Reintroduction of corticosteroid treatment resulted in complete clinical and laboratory remission.

Facial edema as the sole clinical manifestation of dermatomyositis is extremely rare. There have been no previous reports of isolated facial edema in the setting of acute, clinically amyopathic dermatomyositis in adults. A high level of suspicion is required to make the diagnosis in the absence of myopathy and the hallmark cutaneous manifestations of the disease (heliotrope rash, Gottron papules).

## Introduction

A patient presenting with isolated periorbital and facial edema may pose diagnostic challenges. The differential diagnosis includes allergic reactions, soft tissue infections, intracranial venous thrombosis, auto-immune (dermatomyositis (DM), lupus erythematosus) and auto-inflammatory (periodic syndrome) disorders, orbital myositis, and trichinosis. In this setting, it may be difficult to make an accurate clinical diagnosis, and the patient is often treated empirically, pending the results of specific laboratory investigations. We present a case where skin and muscle biopsy established the previously unsuspected diagnosis of DM in a patient presenting acutely with periorbital and facial edema without myopathy. To our knowledge, there have been no previous reports of isolated facial edema in the setting of acute, clinically amyopathic dermatomyositis.

## Case report

A 45-year-old Asian man, unskilled worker, who had lived in Greece for the last three years, presented to the Emergency Department with a three day history of painful facial swelling, pyrexia and vomiting. His medical history was notable only for type 2 diabetes mellitus treated with metformin and gliclazide. He denied recent travel abroad.

**Figure 1.**
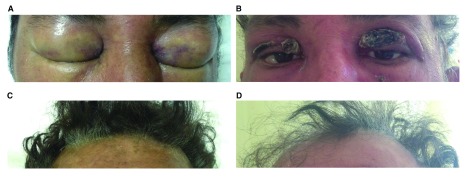
Patient facial appearances before (A,C) and after (B,D) treatment. **A**: Periorbital edema at presentation. The edema was evolving rapidly and the patient was unable to open his eyes. **B**: Subsidence of edema was followed by extensive skin desquamation. Erythema is due to corticosteroid therapy. **C,D**: Development of marked alopecia. Photographs B and D were taken on hospital day 26.

On examination there was bilateral, sensitive to palpation, edema involving the eyelids, nose, malar areas, forehead and anterior part of the scalp, associated with painful cervical lymphadenopathy ( Figure 1A). The rest of the physical examination, including cardiovascular, respiratory, neuromuscular systems and abdomen, was unremarkable - in particular there were no muscle weakness or arthritis, and no skin eruptions on the trunk or extremities. Urgent laboratory investigations showed a picture of inflammation, with neutrophil leukocytosis (17,800/μL, normal 2,500–8,000), increased C-reactive protein levels (323 mg/L, normal <3.5) and elevated erythrocyte sedimentation rate (80 mm/1h, normal <15). Biochemical profile revealed hyperglycemia (glucose 385 mg/dL, normal 70–100) and mild compensated acidosis (serum bicarbonate 14.5 mmol/L, normal 22–29), but was otherwise unremarkable. Notably, serum muscle enzyme levels (creatine phosphokinase, aspartate aminotransferase, alanine aminotransferase, lactic dehydrogenase) were normal. Urinalysis and chest radiogram were normal. Abdominal ultrasound examination revealed cholelithiasis without signs of cholecystitis. The patient was admitted to hospital and, in the absence of a diagnosis, was treated empirically with intravenous ampicillin-sulbactam (3 g q.i.d.), clindamycin (600 mg t.i.d,) dimethindene (4 mg b.i.d. for 3 days, then o.d. for 7 days) ranitidine (50 mg b.i.d.), and methylprednisolone (40 mg t.i.d.). Soluble insulin was administered subcutaneously as required, based on capillary glucose measurements t.i.d.. Blood cultures and screening for infections, including HIV and hepatitis were negative. Computed tomography and magnetic resonance imaging (MRI) of the head showed marked subcutaneous tissue edema of face and cranial vault, while MRI venography was normal.

A skin and muscle biopsy was obtained from the right malar area. The facial edema and cervical lymphadenopathy remitted gradually over the following days in parallel with falling levels of inflammation markers. No abnormalities were detected on repeated clinical assessment of muscle tone, power and tendon reflexes throughout his hospitalization. An attempt to discontinue corticosteroid therapy on hospital day 9 was quickly followed by relapse of facial edema, pyrexia and cervical lymphadenopathy, associated with a new rise of inflammation markers. Discontinuation of antibiotics and reintroduction of methylprednisolone (40 mg I.V. t.i.d, switching to 16 mg p.o. t.i.d after five days) on hospital day 14 led to a new clinical and laboratory remission within five days.

**Figure 2.**
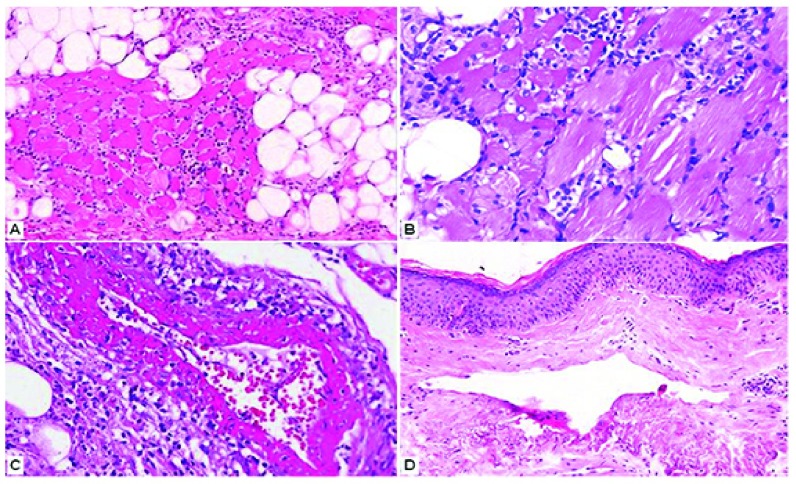
Skin and muscle biopsy. **A, B**: Myositis in the form of a mainly lymphocytic cellular infiltrate of striated muscle fibers with subsequent destruction and regeneration. (Hematoxylin and eosin x250, x400.) **C**: Leukocytoclastic vasculitis (fibrinoid necrosis and nuclear dust) in septal vessels of the subcutaneous tissue. (Hematoxylin and eosin x400.) **D**: Telangiectatic vessels in the reticular dermis, edema and a mild lymphocytic infiltrate. (Hematoxylin and eosin x200.)

Additional investigations showed an elevated antinuclear antibody titer (1:320, normal <1:80), while anti-dsDNA, RNP, Sm, SSA(Ro), SSB(La), Jo1, Mi2 autoantibodies and rheumatoid factor were negative. *Trichinella spiralis* IgG antibodies were also negative. C1-inhibitor levels were normal, while serum C3 levels were low (26.9 mg/dl, normal 88–135) and C4 levels were within normal range. Serum aldolase levels were normal. The muscle biopsy findings were typical of DM, while skin biopsy histology was compatible with, although not diagnostic of DM. ( Figure 2).

By day 33, all laboratory abnormalities had returned to normal. Edema of face and scalp had completely subsided, leaving extensive desquamation and alopecia of involved areas ( Figure 1B–D). The patient was discharged taking methylprednisolone 32 mg daily, with arrangements for rheumatology outpatient follow-up. He declined further investigations to exclude an underlying malignancy.

## Discussion

In its typical form, DM presents with the unique combination of inflammatory myopathy and characteristic cutaneous lesions (heliotrope periorbital edema, Gottron papules and sign, shawl and V-sign, holster sign). Internal organs (lungs, heart, and gastrointestinal tract) may become involved, contributing to the significant morbidity and mortality of patients with DM who are not diagnosed and treated at an early stage. The importance of timely diagnosis is enhanced by the fact that approximately 15% of adult patients with newly diagnosed DM either have a preexisting malignancy or will develop one within a few years following diagnosis ^1, 2^ When clinical or laboratory manifestations of myopathy (proximal muscle weakness or elevated muscle enzyme levels in the serum) are absent, as was the case with our patient, DM may elude diagnosis. This can occur in the early stages of the illness, when muscle involvement may not yet be evident (“pre-myopathic DM”). The term “amyopathic DM” (synonym: dermatomyositis-sine-myositis) is usually reserved for patients with the hallmark cutaneous manifestations or histological findings of DM who have normal serum muscle enzyme levels and no muscle weakness for at least 6 months after the initial diagnosis (10–20% of all cases of DM, may be higher in Asian populations) ^3^. A limitation of the present report is the lack of long-term outcome information because the patient was lost to follow-up.

Although cutaneous manifestations of DM commonly involve the face, isolated facial edema as the sole clinical sign of disease is an extremely rare occurrence. Such an atypical presentation can lead to significant delays in diagnosis. Hall *et al*. reported a case where DM was diagnosed only when the patient was hospitalized for dysphagia, six months after his initial presentation with severe periorbital edema ^4^. Subcutaneous edema, usually localized or regional, is being increasingly recognized as a manifestation of DM and appears to be associated with more aggressive disease ^5, 6^. The presumed pathophysiological mechanism involves increased permeability of capillaries caused by complement-mediated microvascular endothelial damage. In DM, putative antibodies directed against antigens of capillary endothelium activate the classical complement pathway, resulting in deposition of membrane attack complex on capillary endothelial cells early in the inflammatory process ^7^. This is in keeping with the low serum complement C3 levels present in our case. Other, non-immunological mechanisms might be contributing to increased capillary permeability: Levels of vascular endothelial growth factor (VEGF), which induces hyperpermeability by a direct action on endothelial cells, have been found to be increased in muscle tissue and plasma of patients with early-phase DM ^8, 9^.

In conclusion, DM should be included in the differential diagnosis of patients presenting acutely with isolated facial edema of unknown cause, even when other clinical and laboratory manifestations of the disease are absent. An aggressive diagnostic approach with skin and muscle biopsy may enable early diagnosis and treatment of this potentially fatal disease.

## Consent

Written informed consent for publication of his clinical details and clinical images was obtained from the patient.
