# Supplementary material for: Case Report: Clinically amyopathic dermatomyositis presenting acutely with isolated facial edema
Source: F1000Res. 2018 Mar 26;7:60. Originally published 2018 Jan 16. [Version 2] doi: 10.12688/f1000research.13604.2 (PMC5883386; doi:10.12688/f1000research.13604.2)
Supplement: Peer review history

# Peer review: Referee response for version 2

**DOI:** 10.5256/f1000research.15685.r32410

**Published:** 2018-04-03

**Authors:** Lyubomir A. Dourmishev

**Affiliations:** 1 Department of Dermatology and Venereology, Medical University, Sofia, Sofia, Bulgaria

No further remarks.

I have read this submission. I believe that I have an appropriate level of expertise to confirm that it is of an acceptable scientific standard.

# Peer review: Referee response for version 1

**DOI:** 10.5256/f1000research.14778.r31365

**Published:** 2018-03-05

**Authors:** Albert Selva-O'Callaghan

**Affiliations:** 1 Department of Internal Medicine, Autonomous University of Barcelona, Barcelona, Spain

The authors report the case of a patient with periorbital and facial oedema in whom muscle biopsy allowed the diagnosis of myositis (probably DM). The skin biopsy in this case is not specific, absence of mucine and leukocytoclastic vasculitis are not typical of DM. This reviewer, nevertheless, agree that the most probably diagnosis is DM. In this case it would be appropriate to define it as a Clinically Amyopathic DM.

 Determination of other specific autoantibodies would have been of help: anti-TIF1gamma, anti-PL12, anti-PL7, anti-SRP, anti-MDA5, or anti-NXP2. 

 In opinion of the reviewer, this presentation is not so rare.

 Please comment or modify all these points.

I have read this submission. I believe that I have an appropriate level of expertise to confirm that it is of an acceptable scientific standard.

# Response

**Published:** 2018-03-22

**Authors:** Efthymia Pappa

**Affiliations:** Amalia Fleming General Hospital, Greece

We thank Dr. Selva-O’Callaghan for critically reviewing our manuscript. Our response is as follows:

1. We have used the term “clinically amyopathic dermatomyositis” in the revised version, as suggested by both reviewers. We have also clarified that the skin biopsy findings were compatible with but not diagnostic of DM.
2. Among the autoantibodies specific for myositis, we only had access to anti-Mi2 and anti-Jo1. Anti-Mi2 tends to be positive in classical DM with typical skin manifestations and high muscle enzyme levels, both of which were absent in our case. Positive anti-Mi2 was reported in 38.8% of DM patients from the Indian subcontinent (P Srivastava *et al*. Rheumatol Int 2016, DOI 10.1007/s00296-016-3494-3). In the same ethnic group, Jo1 was detected in 5.4% of DM patients.
3. Although the face is frequently the only area affected in dermatomyositis, particularly in the early stages, we could find no published cases of adult DM  presenting with isolated facial edema without rash or muscle enzyme abnormalities. 

# Peer review: Referee response for version 1

**DOI:** 10.5256/f1000research.14778.r30305

**Published:** 2018-02-15

**Authors:** Lyubomir A. Dourmishev

**Affiliations:** 1 Department of Dermatology and Venereology, Medical University, Sofia, Sofia, Bulgaria

The case report presents the diversity of clinical variants in autoimmune inflammatory myopathies. Clinical case is present briefly, but the information is sufficient.

 My critical remarks are:

1. It is not clear how the authors clinically assess the muscle weakness;
2. Authors state creatine phosphokinase, aspartate aminotransferase and lactic dehydrogenase are in normal ranges. What about alanine aminotransferase and aldolase?
3. I would suppose the term clinical amyopathic dermatomyositis, since muscle biopsy shows definite features of myositis and thus is the key evidence for diagnosis.
4. It is not indicated the place of muscle biopsy.
5. I would recommend discussing the orbital myositis as a possible differential diagnosis.
6. Investigation of anti-MDA5 and may have value in this case.

In conclusion article is interesting and after some corrections will be useful for variety of medical specialists.

I have read this submission. I believe that I have an appropriate level of expertise to confirm that it is of an acceptable scientific standard, however I have significant reservations, as outlined above.

# Response

**Published:** 2018-03-22

**Authors:** Efthymia Pappa

**Affiliations:** Amalia Fleming General Hospital, Greece

We thank Professor Dourmishev for his constructive comments, which we took into account in revising the manuscript. Our detailed response to his remarks is as follows:

1. Muscle weakness was assessed by clinical examination. No abnormalities were detected on repeated clinical assessment of muscle tone, power and tendon reflexes. A relevant passage has been included in the revised text.
2. Serum alanine aminotransferase levels were also normal. This has been stated in the revised text. Aldolase levels were normal – this is stated in the original text (paragraph 4, line 5 of case report section).
3. We have used the more accurate term “Clinically amyopathic dermatomyositis” as suggested by the reviewer.
4. We have indicated the site of biopsy (right malar area) in the revised text.
5. Orbital myositis has been included in the differential diagnosis as suggested.
6. We only had access to Jo1 and Mi2 autoantibodies at that time, and therefore no anti-MDA5 or other autoantibodies of potential value in DM could be looked for. We note, however, the low sensitivity of such antibodies. Only 6.9% of DM patients in a recent USA study had positive anti-MDA5, and most of these had overt myopathy (JC Hall *et al*. Arthritis Care Res. (Hoboken) 2013; 65: 1307-15).
